# Analyzing Patient Complaints in Web-Based Reviews of Private Hospitals in Selangor, Malaysia, Using Large Language Model–Assisted Content Analysis: Mixed Methods Study

**DOI:** 10.2196/69075

**Published:** 2025-06-27

**Authors:** Muhammad Hafiz Sulaiman, Nora Muda, Fatimah Abdul Razak

**Affiliations:** 1 Department of Mathematical Sciences Faculty of Science and Technology National University of Malaysia Bangi Malaysia; 2 Quality Unit Clinical Management Section Hospital Sultan Idris Shah Serdang Malaysia

**Keywords:** large language model, hospital quality, patient satisfaction, big data, web-based review

## Abstract

**Background:**

Large language model (LLM)–assisted content analysis (LACA) is a modification of traditional content analysis, leveraging the LLM to codevelop codebooks and automatically assign thematic codes to a web-based reviews dataset.

**Objective:**

This study aims to develop and validate the use of LACA for analyzing hospital web-based reviews and to identify themes of issues from web-based reviews using this method.

**Methods:**

Web-based reviews for 53 private hospitals in Selangor, Malaysia, were acquired. Fake reviews were filtered out using natural language processing and machine learning algorithms trained on yelp.com validated datasets. GPT-4o mini model application programming interface (API) was then applied to filter out reviews without any quality issues. In total, 200 of the remaining reviews were randomly extracted and fed into the GPT-4o mini model API to produce a codebook validated through parallel human-LLM coding to establish interrater reliability. The codebook was then used to code (label) all reviews in the dataset. The thematic codes were then summarized into themes using factor analysis to increase interpretability.

**Results:**

A total of 14,938 web-based reviews were acquired, of which 1121 (9.3%) were fake, 1279 (12%) contained negative sentiments, and 9635 (88%) did not contain any negative sentiment. GPT-4o mini model subsequently inducted 41 thematic codes together with their definitions. Average human-GPT interrater reliability is perfect (κ=0.81). Factor analysis identified 6 interpretable latent factors: “Service and Communication Effectiveness,” “Clinical Care and Patient Experience,” “Facilities and Amenities Quality,” “Appointment and Patient Flow,” “Financial and Insurance Management,” and “Patient Rights and Accessibility.” The cumulative explained variance for the six factors is 0.74, and Cronbach α is between 0.88 and 0.97 (good and excellent) for all factors except factor 6 (0.61: questionable). The factors identified follow a global pattern of issues identified from the literature.

**Conclusions:**

A data collection and processing pipeline consisting of Python Selenium, the GPT-4o mini model API, and a factor analysis module can support valid and reliable thematic analysis. Despite the potential for collection and information bias in web-based reviews, LACA of web-based reviews is cost-effective, time-efficient, and can be performed in real time, helping hospital managers develop hypotheses for further investigations promptly.

## Introduction

### Quality Improvement Activities in Hospitals

Getting feedback from patients and families is important to continuously improve patient care and ensure patient and family satisfaction within health care settings [1,2]. Patient and family satisfaction is crucial for repeat visits and the economic sustainability of the health care provider; therefore, management teams need to empathize with their patients’ and families’ understandings, feelings, and behaviors in order to thrive in a competitive health care market.

### Web-Based Reviews

The use of traditional surveys like SERVQUAL to measure quality in Malaysian health care is documented by Butt and de Run [3], Aliman and Mohamad [4], and Abd et al [5]. Observations, formal interviews, and surveys such as SERVQUAL are standard methods for collecting feedback from patients and families, but all require a significant amount of time for data collection [6,7]. Since these methods are resource-intensive, obtaining unbiased results will require researchers to invest more time and money, otherwise, the number of respondents is limited, which could lead to low study power and false negative results. Additionally, ethnographic studies often fall short in their ability to observe patients before, during, and after hospital stays due to privacy concerns from both the clinicians’ side and the patients’ side [8].

The use of web-based reviews to gather feedback on patients’ experiences and opinions can help hospital managers address the limitations mentioned above. Unlike ethnographic studies and interviews, web-based reviews by patients and families have no spatial limitations, meaning that patients and families can share their feelings, experiences, and opinions throughout their entire journey—before, during, and after their hospital stay or visit. Web-based reviews are also readily available on the internet, and with the aid of a large language model (LLM)–assisted content analysis (LACA), we can include a larger number of respondents, thereby reducing the risk of type II error (false negatives) caused by an insufficient sample size.

Ranard et al [9] mentioned the advantages of using web-based reviews for hospital quality improvements, including the diversity of domains reported in web-based reviews. Traditional surveys such as Hospital Consumer Assessment of Healthcare Providers and Systems (HCAHPS) have fixed domains in which the questions are based and have origins that derive from 1995. Since patients’ indications and experience for hospitalization have changed greatly, using a fixed set of surveys would set a barrier to fully understanding current patients’ needs. His study found 12 web-based review domains not otherwise reflected in HCAHPS.

Rahim et al [10], in their paper, however, raised the need for health care organizations to change in accordance with Industrial Revolution 4.0 by using web-based reviews to understand patients’ and families’ interests, desires, and values. He too mentioned that the use of traditional surveys like HCAHPS and SERVQUAL is restrictive in the ways that these surveys are fixed, time-intensive, lengthy, fail to identify the causes of concern, and are subject to response and selection bias. The author suggested using web-based reviews, such as Facebook web-based reviews on hospitals’ pages, as new sources for quality monitoring in hospitals and using supervised machine learning (ML) to train ML models to classify these reviews into SERVQUAL domains.

### Publicly Available Web-Based Data

The exponential growth of digital communication channels has transformed health care feedback mechanisms in Malaysia and globally. Malaysia’s internet adoption rate reached 96.8% in 2023 [11], with social media users aged 18 years and older exceeding 24.8 million, representing 99.8% of the population [12]. This digital transformation has generated substantial health care–related user-generated content.

While global data use was projected to grow from 33 ZB in 2018 to 175 ZB by 2025 [13], Malaysia’s health care sector has seen its own surge in digital footprint, with an estimated 54.7% of all patients using the internet to search for health information [14] and 91.2% health care workers have good eHealth literacy [15]. Web-based data reflecting patients’ experiences in Malaysian health care settings is increasingly available through various digital channels, including social media platforms, hospital review websites, health care forums, and patient blogs. The Health White Paper for Malaysia further emphasizes the importance of leveraging these digital data sources for health care quality improvement [16].

### Study Designs

This study is exploratory in nature, and our research questions are as follows: (1) How to apply LACA satisfactorily on hospital web-based reviews? (2) What are the themes of issues identified by LACA on hospital web-based reviews? The purposes of this study are (1) to develop and recommend a method for analyzing hospital web-based reviews to serve as an alternative to direct observations or interviews on patients and families in hospital settings and (2) to then identify themes of current issues in private hospitals in Selangor, Malaysia. For these purposes, we developed two hypotheses: (1) LACA developed based on our methods produces satisfactory coding works equivalent to a human coder with Cohen κ>0.80; (2) the themes identified from factor analysis produce a Cronbach α>0.70 on all factors with interpretable items.

The population of the study is patients or families who have posted their reviews on a web-based review platform for private hospitals in the state of Selangor, Malaysia, from January 1, 2023, to December 31, 2023. All 53 private hospitals in the state were included in this study. This population was chosen because researchers and experts involved in this study have good knowledge of local private hospitals as compared to hospitals located somewhere else, therefore, enabling them to contribute to the qualitative inputs needed in this research. This study includes all reviews posted on a web-based review platform for all private hospitals inside Selangor using universal sampling so that we could get as much diversity of reviews as possible, a point of advantage over traditional observations or interviews.

The web-based reviews are then filtered to exclude reviews that are not accompanied by any comments. Since web-based reviews are subject to manipulation by hospitals, we also exclude fake reviews using natural language processing (NLP) and ML algorithms to make sure that the results represent the real patients’ suggestions, opinions, and experiences. Detailed explanations of this method are discussed below.

## Methods

### Thematic Analysis

Thematic analysis is a qualitative research method used to identify, analyze, and report patterns or themes within data. It begins with researchers immersing themselves in the data to gain a deep understanding, which involves repeatedly reading and reviewing the material. Following this, they generate initial codes by systematically tagging relevant sections of the data with short labels that capture key aspects. These codes are then organized into potential themes—broader patterns that reflect significant features related to the research question.

Researchers review and refine the themes created to ensure they accurately represent the data and fit together cohesively. Each theme is clearly defined and named, and the final step involves writing up the findings to provide a comprehensive interpretation of the data, weaving together the themes to offer insightful conclusions. Thematic analysis is valued for its flexibility and ability to uncover patterns within complex qualitative data. Most of the thematic analysis framework used in this study is based on Braun and Clarke [17].

### Inductive Coding in Thematic Analysis

Inductive coding in thematic analysis is a process where researchers develop codes directly from the data, rather than applying predetermined categories or theoretical frameworks (which apply in deductive coding). It begins with a thorough examination of the data to gain an in-depth understanding. Researchers then identify significant segments of text and create codes based on the content and meaning of these segments. These initial codes are descriptive and reflect the language and concepts used by the participants.

As coding progresses, similar codes are grouped together to form broader themes, which emerge naturally from the data itself. This approach allows for a more grounded analysis, as themes are developed from the participants’ perspectives rather than imposed by external theories. The themes are then reviewed and refined to ensure they accurately represent the data and provide a coherent interpretation. Inductive coding is particularly useful for exploring new research areas and gaining insights that are deeply rooted in the data [18].

### LLM

Traditional methods of analyzing text data from surveys and reviews often pose significant challenges, including time-consuming manual processes and resource-intensive endeavors [19]. Moreover, the sheer volume and unstructured nature of textual data available on the internet further exacerbate the complexity of analyzing and extracting actionable insights using conventional methodologies. Given the overwhelming amount of data deriving from web-based review platforms, attention is increasingly turning toward automated content analysis instead of pure qualitative content analysis [20].

With the emergence of LLMs, such as the GPT-4o mini model, health care institutions now possess a powerful tool to navigate and extract valuable insights from the vast expanse of unstructured text data available on the web. Hassani et al [21] confirm the fact that text mining in big data analytics is emerging as a powerful tool for harnessing the power of unstructured textual data by analyzing it to extract new knowledge and identify significant patterns and correlations hidden in the data.

LLMs are sophisticated artificial intelligence (AI) models trained on large corpora of text data [22], enabling them to understand and generate human-like language with remarkable accuracy and fluency. GPT architecture incorporates attention mechanisms and feed-forward neural networks to predict the next word in a sequence, which has improved LLM functionality significantly [23]. Leveraging advanced NLP techniques, LLMs excel in tasks such as text summarization, thematic analysis [24], and sentiment analysis [25], making them well-suited for analyzing qualitative data in health care contexts.

LLM is increasingly experimented with in the health care industry. For example, recent studies were conducted to see the impact of LLM in drug discovery [26], extraction of medical notes [27], prediction of diagnosis-related groups [28], and diagnostics [29]. A study by Lin and Kuo [30] highlighted the huge opportunities to leverage LLM in clinical decision-making systems. The advancement of LLM is parallel to the world’s movements toward using AI in health care. The Ministry of Health, Malaysia [31], for example, mentioned the use of AI and big data as essential tools for improved health care delivery in the near future. The Ministry of Health Malaysia also made digitalization, advanced data analytics, and AI as the main agenda for the country’s health reformation [16].

The use of LLMs in quality improvement activities offers several notable advantages over traditional methodologies. First, LLMs enable the automated processing and analysis of large volumes of unstructured text data, significantly reducing the time and resources required for data collection and analysis. This scalability allows health care institutions to extract insights from diverse sources of patient feedback in a timely and efficient manner, facilitating rapid response to emerging trends or issues.

Additionally, LLMs can identify nuanced patterns, sentiments, and themes within diverse textual data, providing deeper insights into patients’ perceptions of health care quality and identifying areas for improvement that may have been overlooked using manual methods.

Furthermore, the integration of LLM-driven insights into quality improvement initiatives has the potential to enhance the patient-centeredness of health care delivery. By capturing and analyzing patients’ experiences, opinions, and preferences as expressed in their own words, health care institutions can gain a more comprehensive understanding of patient needs and priorities. This patient-centric approach enables tailored quality improvement interventions that address specific patient concerns, ultimately leading to improved patient satisfaction and outcomes.

### LACA

LACA is a term coined by Chew et al [32] that describes the use of LLMs to enhance and streamline qualitative content analysis. Researchers begin by inputting qualitative data—such as text from interviews or documents—into an LLM, which processes and summarizes the content to provide an initial understanding. The LLM aids in coding and categorizing the text by suggesting themes and patterns based on its advanced NLP capabilities. This helps in identifying and organizing key topics and underlying themes more efficiently [33].

LLMs offer several advantages in the coding process, starting with consistency and standardization. By using sophisticated algorithms, they apply uniform criteria across datasets, minimizing variability and subjectivity that can arise from different human coders. This ensures coding reliability, especially when working with large datasets, where human inconsistencies can become more pronounced. Such standardization is crucial for maintaining high-quality and reliable results [24,34].

Another key benefit of LLMs is their scalability. These models can process and analyze vast amounts of text data much more quickly than manual methods, addressing significant resource constraints in qualitative research. The ability to handle large datasets efficiently makes LLMs an ideal solution for large-scale projects, where traditional methods may not be feasible due to time and personnel limitations [21].

LLMs also improve efficiency by automating the coding process, reducing the time required for annotation compared to manual coding. This can lead to substantial time savings, although the exact extent of these savings still requires further empirical investigation. The automation of this process helps streamline workflows and allows researchers to focus on other aspects of their projects [35].

In addition to efficiency, LLMs contribute to error reduction by adhering to predefined coding rules. This minimizes inconsistencies often caused by human fatigue or differences in interpretation. While LLMs can reduce errors, careful validation against human coding is still necessary to ensure accuracy and reliability in the results [36,37].

The potential for replicability and cost-effectiveness is another strength of LLM-based coding. When using the same models and algorithms, LLMs can produce more consistent results, making it easier to replicate studies or analyses. However, long-term reproducibility remains an area of ongoing research, as models and datasets evolve over time [38,39]. In terms of cost-effectiveness, LLM-assisted coding can reduce the need for large human teams, particularly for large-scale projects. However, it is important to consider the initial implementation costs and the resources required for model training. Despite these costs, the long-term savings in human resources and time can make LLM-based coding a more economical solution for large research projects [40,41].

Finally, LLMs have the capability for complex pattern detection. Trained on extensive datasets, they can identify nuanced patterns that manual coding might overlook. While this ability enhances the depth of analysis, it also requires rigorous validation to ensure that the patterns detected are meaningful and accurate [42,43]. Each of these factors—consistency, scalability, efficiency, error reduction, replicability, cost-effectiveness, and complex pattern detection—demonstrates the potential of LLMs to significantly enhance coding processes in qualitative research.

LACA uses an LLM, and in this study, is specifically using a GPT in which prompts were transformed to trigger the model to generate responses, that is, codes or attribute labels. Other than this GPT method, latent Dirichlet allocation is widely used to classify documents into topics. GPT is advantageous over latent Dirichlet allocation because GPT is context-aware and able to directly produce textual descriptions [44]. In summary, LACA offers improved accuracy, efficiency, and scalability, making it a powerful tool for handling extensive and intricate content analysis tasks.

### Ethical Considerations

As this study involves secondary analysis of publicly available web-based reviews, which were anonymized and deidentified during the results visualization stage, no ethical review/approval was needed, per the Malaysian Medical Review and Ethics Committee (MREC) Ministry of Health guidelines [45]. No personal or hospital identifiers were associated with the reviews in the final results. Data processing was done only by authorized researchers, while data filtering was performed to ensure the exclusion of fake reviews using NLP and ML algorithms, which were trained on publicly available datasets such as Yelp.com. Protections were implemented during the presentation of results to minimize any potential risk of identifying individuals or revealing sensitive information. The study methodology adheres to ethical standards concerning the use of publicly available web-based content for research purposes.

### Flow of the Study

**Figure 1:**
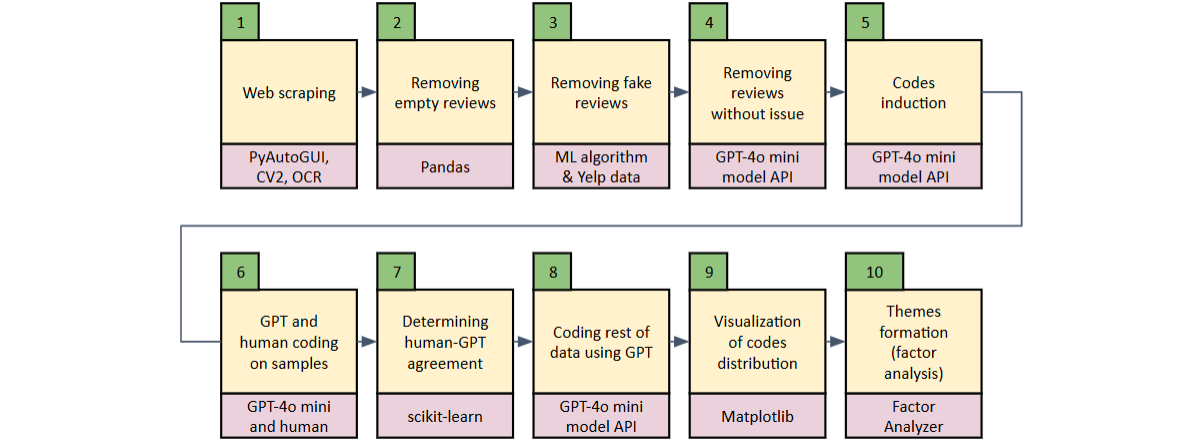
Flow of research (summarized).

The flow of the study is shown in Figure 1.

#### Web Scraping

We begin our research by getting a list of private hospitals in the state of Selangor sourced from the Ministry of Health Malaysia’s website. The list was used to search for the hospital’s name on Google Search. This allowed us to locate Google Reviews page for each of these hospitals. We develop a program using the Python PyAutoGUI module to automate data scraping. Ratings were extracted using computer vision (CV2) since star ratings in Google Reviews are presented in .jpg format and not in text format. Optical character recognition techniques were used to detect and locate the word “Newest” on tabs so that we could click the “Newest” button and ensure that the data were sorted from newest to oldest. There were many reviews written too long, so part of the reviews were hidden and readers had to click on the “more” button to reveal the hidden message. We automated the process of recognizing the “more” button by using CV2 and automatically expanding the text using the PyAutoGUI click function. The process of copying all text to the computer’s memory is done using PyAutoGUI’s select, scroll, and copy functions. The copied text was stored in a .txt file with a highly specific separator between reviews.

#### Removing Empty Reviews

Web-based reviews that are not followed by comments are removed, since we are doing qualitative data analysis on texts. This is done using the Python Pandas module by excluding documents with empty text.

#### Removing Fake Reviews

Since web-based reviews are susceptible to manipulation by individuals from the same institution (in the case of fake positive reviews) or competitors (in the case of fake negative reviews), these fake reviews do not represent real opinions, experiences, and suggestions. To do this, a program was developed based on previous studies [46-49] that incorporates NLP methodologies with ML algorithms. All data for training and testing the algorithm was acquired from yelp.com, a web-based review platform for hotels, restaurants, and hospitals, among others, that separates fake reviews from real reviews. The dataset was fed into a natural language preprocessing pipeline, which included the process of standardization, punctuation removal, numerical removal, tokenization, stop word removal, and formation of trigrams. Each trigram is now forming a single column in a matrix of term frequency-inverse document frequency, a vector matrix that becomes an input for a support vector machine and logistic regression ML algorithm. The ML model was trained and tested using the preprocessed yelp.com dataset and achieved a precision of 0.87, a recall of 0.89, a high *F*_1_-score, and an accuracy of 0.88. Using the same NLP pipeline, each of our hospital review documents is preprocessed and then transformed into vector form (term frequency-inverse document frequency) before being fed into our validated ML model to filter out as many fake reviews as possible.

#### Removing Comments Without Issue

A comment can have good (positive) sentiment, bad (negative) sentiment, or can have both. This study focuses on identifying issues (negative sentiment) related to quality in hospitals. A previous study on bias in web-based reviews by Roh and Yang [50] showed that bad reviews are the most meaningful to help readers make decisions about a hospital. Other than that, we also tried to avoid having to make tuples for each label or code, that is, having to label or code (“communication,” “negative”) and (“communication,” “positive”) instead of just “communication” because tuples will produce double the number of variables, a phenomenon seen in the paper by Zaman et al [51]. A long list of variables also makes it more difficult during the theme formation (dimension reduction) phase. We maintain reviews containing negative sentiment and filter out the remaining by calling the GPT-4o mini model application programming interface (API) to respond “Yes” if a review contains issues and “No” otherwise. Full prompts are given in Multimedia Appendix 1.

#### Codes Induction

This stage begins with extracting issues present in each review. GPT-4o mini model API was used to identify, summarize, and list issues raised by each customer based on their web-based customer review. Then the next step involves randomly sampling 200 of these extracts to feed the GPT-4o mini model to produce lists of codes together with their definitions (codebook). A total of 5 iterations were performed to create a comprehensive list of codes using the GPT-4o mini model API calls. The final code list was reviewed by health care management experts and used as a codebook to code issues in the dataset. Full prompts are provided in Multimedia Appendices 2 and 3.

#### GPT and Human Sample Coding

A random 200 reviews were selected. GPT-4o mini model API was tasked to use the codebook and label the 200 reviews. A human researcher was also tasked to label the same 200 reviews. Both GPT and human coders were instructed to code each review by iterating each item in the codebook and answer 0 if the item is not an issue in the review, 1 if the item is a small issue, 2 if the item is a moderate issue, 3 if the item is a serious issue, and 4 if the item is an extremely serious issue in the review (Multimedia Appendix 4). If there are *n*_i_ items or codes in the codebook, the number of API calls will be 200*n*_i_. Interrater reliability was evaluated between a human coder and a GPT coder across the 200 reviews or documents using Cohen κ, a statistical measure that accounts for chance agreement.

#### Determining GPT-Human Agreement

At this stage, we will have a matrix of 200×*n*_i_ where the rows are reviews or documents and the columns are item variables, *i*. Each cell contains integers from 0 (indicating no issue) to 4 (indicating extremely serious issue). Interrater reliability is a crucial aspect of ensuring the reliability of coding and categorization in our research. We do this by converting the scalar data type to binary (0 if no issue, and 1 if there is at least one issue related to the thematic code). Cohen κ was calculated between the GPT and the human coder for each document. The average κ score across the 200 documents was calculated to assess overall agreement. An average Cohen κ of more than 0.8 is acceptable to proceed to the next stage.

#### Coding the Rest of the Data Using GPT

As GPT coding tasks are proven to be reliable, the process of coding (described above) can then be continued solely by the GPT-4o mini model on the rest of our data. This way, we can label all our current data (and other data in the future) easily without the need for human coders. This is the advantage of our current method as compared to manually coding the web-based reviews.

#### Visualization of Codes Distribution

During this stage, we visualized the code distribution in our dataset, including the prevalence of each of the codes. Co-occurrences of codes are visualized through heat maps of correlation between individual codes. Pearson correlation was conducted to see the strength of the association between thematic codes.

#### Themes Formation by Factor Analysis

Thematic codes used to label the review were reduced to single-digit latent factors. Latent factors are essentially the underlying variables that explain the patterns of correlations among observed variables. The formation of single-digit themes is important so that we can focus our efforts on these themes. Although this process can be done manually through rearrangements of codes into themes, we automate the process so that any future analysis will also be done automatically for efficiency. The use of factor analysis to reduce attributes or codes into themes is documented by Sovacool [52]. We decided to include or exclude factors based on cumulative explained variance, Cronbach α [53], and qualitative assessment—content validity [54].

Overall, the methodology involves a systematic approach to collect, integrate, analyze, and interpret data from web-based reviews to understand the factors influencing demand for private hospitals in Selangor. Advanced techniques like ML and NLP were used to filter out fake reviews and extract meaningful insights from large datasets.

## Results

### Overview

Exactly 14,938 Google Review data points were scraped by our program, developed using a graphical user interface. These data include 53 private hospitals in the state of Selangor. The data collected consists of data from one year prior to the date the data were collected (January 2023-December 2023). Of the 14,938 data collected, a total of 12,035 (81%) evaluations were accompanied by comments, while 2903 (19%) evaluations were not accompanied by comments. Among all reviews with comments, 1121 (9.3%) reviews were fake and excluded from the data. There are 1279 evaluations that have issues (negative sentiments), and 9635 evaluations do not have issues (positive or neutral sentiments). The full list of thematic codes generated by LLM and its definition, that is, the codebook, can be accessed in Multimedia Appendix 5. An example of actual web-based reviews and the codes labeled to them by LLM is shown in Table 1 and more in Multimedia Appendix 4.

Cohen κ revealed a perfect level of agreement between the human and GPT coders. Across the 200 random reviews or documents, the lowest κ score between human and GPT coder was 0.44, and the highest κ score was 1.00. The average Cohen κ score was found to be exceptionally high (0.81), indicating a very strong level of agreement beyond what would be expected by chance. These results demonstrate that the GPT coder exhibited a high degree of consistency in its coding, with minimal discrepancies across the dataset. As GPT coding tasks are validated, the process of coding was then continued solely by the GPT-4o mini model to the rest of our 1279 data points (reviews), which gives us the following finding.

**Table 1:** Example of actual web-based reviews and the themes assigned by LLM^a^.

No.	Actual web-based review	Codes assigned by LLM
1	Staff R****i very helpful but waiting time is too long^1^. 2 hours though. I’m patient no. 4.	1. Waiting time
2	Very friendly staff. We were the regular there since my new born daughter always went there check up and vaccines. It’s fine. But when comes to serious illness, something emergency^2,3^, they are really lack of experienced staff^1,4^.	1. Work load 2. Emergency services 3. Patient safety and hygiene 4. Doctor’s qualification and doctor’s change
3	On 2nd Feb 2023 this S**a nurse said will check for me for an available appointment for this specialist doctor, whom I want to see. She didn’t revert back to me at all^1,2,3,4^. I called again on 28th February this nurse A***a said my appointment was slot on 23rd March @ 5 pm so on 22nd March I call up to confirmed and they said my name was not in the system and I have been waited for almost 1 month. Nurse M*****h help me to rebooked but didn’t inform me that the date has been postponed to 24th March^1,2^ instead she told me is 3 pm so I thought is in 23rd March at 3 pm. Such a private hospital so incompetent and inefficient^4^ the nurses here. I just wants to make an appointment as this doctor specialist is always full. Simple tasks can’t do it well. How you expect people will come to this hospital?	1. Communication 2. Staff responsiveness 3. Staff attitude 4. Inefficient and disorganized processes

^a^LLM: large language model.

### Distribution of Themes Found From Web-Based Reviews

The most common themes based on web-based reviews include the themes of “Service Quality and Professionalism” (n=511), “Communication” (n=506), “Waiting Time” (n=382), “Staff Attitude” (n=284), and the theme of “Responsiveness” (n=192). The least common themes based on web-based reviews include the themes of “Workload” (n=9), “Insurance Billing Errors” (n=9), “Communication Barriers” (n=10), “Comparison Between Races” (n=11), and the theme of “Support for Breastfeeding Mothers” (n=11). The complete list of distributions can be seen in the distribution in Multimedia Appendix 6.

### Correlation Between Thematic Codes

**Figure 2:**
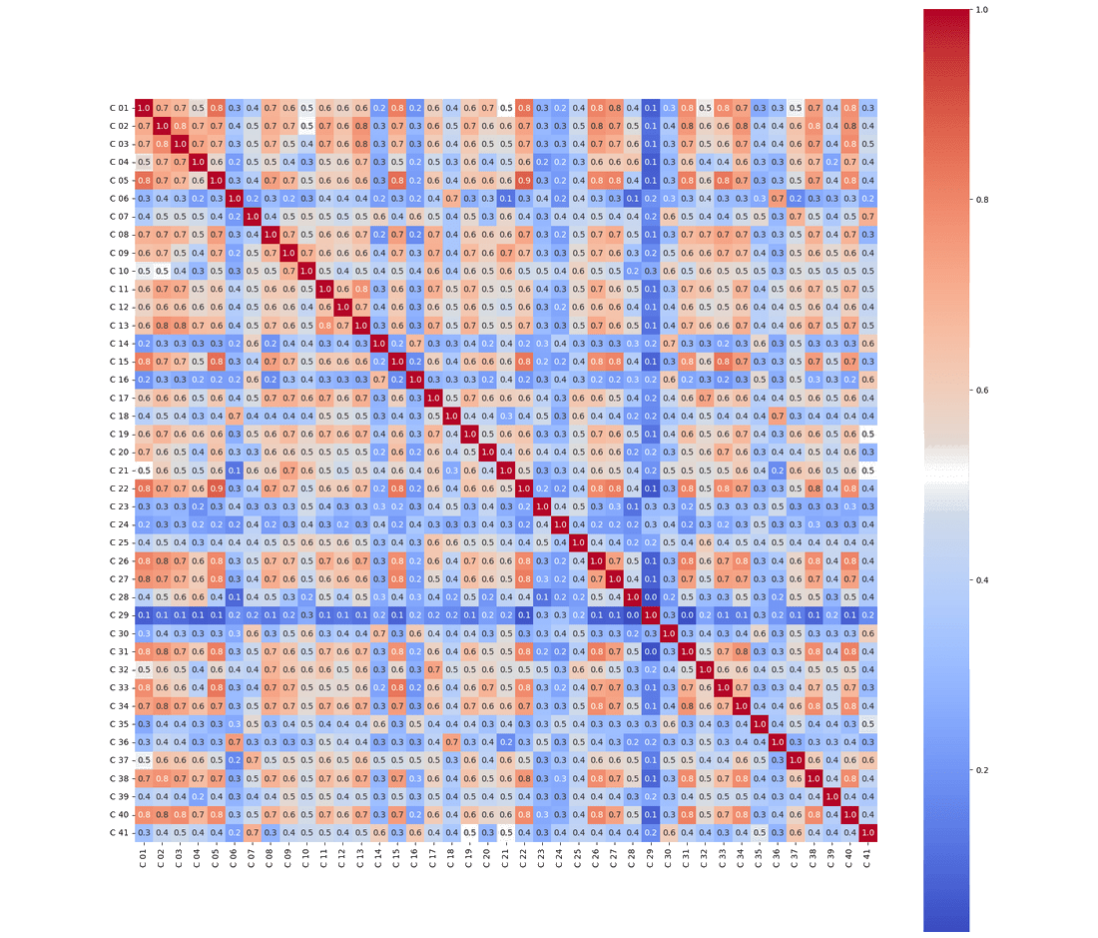
Heat map of correlations between thematic codes.

The correlation was done between 2 thematic codes at a time to observe the relationship between them (please refer to Multimedia Appendix 5 for codes definition). We used Pearson correlation, and our analysis found correlation ranges from –0.0 to 0.9 between each thematic code (Figure 2). The correlation between thematic codes is shown in the correlation heat map below. After the Kaiser-Meyer-Olkin test (0.98) and the Bartlett test of sphericity (*P*<.05), we accepted our data size as adequate and the code matrix as significantly different from the identity matrix and proceeded to the next step—factor analysis.

The heat map shows the correlation between thematic codes. As we can see, most thematic codes have a majority of red boxes, indicating a high correlation with most of the other thematic codes. There are, however, thematic codes that have the majority of blue boxes indicating low correlation with most of the other thematic codes, including these thematic codes: Financial Concerns, Facility Maintenance, Cafeteria and Facilities, Room and Amenities, Patient Privacy, Accessibility for Individuals with Disabilities, Breastfeeding Support, Lift and Equipment Issues, and Insurance Billing Mistakes. The heat map also shows some columns having similar patterns (of red and blue boxes), for example, columns 26, 27, 31, and 33, indicating that they measure an item inside the same theme and should probably be included under the same latent factor when factor analysis is done.

### Factor Analysis

**Figure 3:**
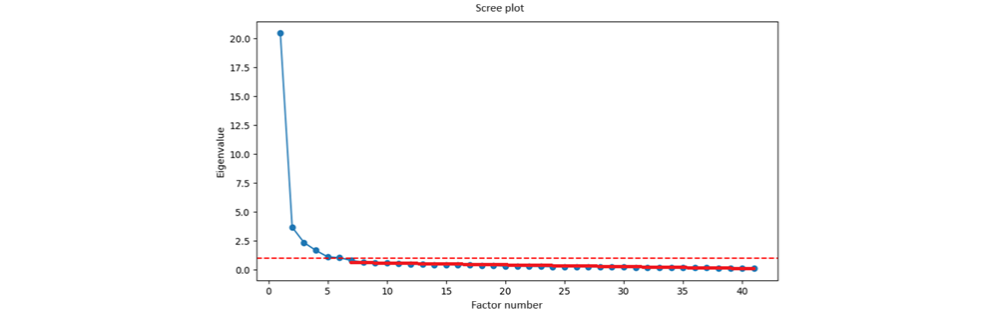
Scree plot of eigenvalue against factor number.

We were using the Factor Analyzer module from Python to derive latent factors from our variables. We determined the appropriate number of latent factors based on eigenvalues and the scree test [55]. Based on the total number of factors with eigenvalues more than 1, the total number of factors to include is 6. Using the scree test, where a straight line is drawn from the least eigenvalue to the largest eigenvalue, the suggested number of factors is also 6 (Figure 3). The Cumulative Explained Variance was 0.74 for the first 6 factors (factors with eigenvalues more than 1). For the full list of cumulative explained variance, please refer to Multimedia Appendix 7. Latent factors, together with their suggested factor labels, codes, factor loadings, and Cronbach α, are summarized in Table 2.

**Table 2:** Latent factors together with their suggested factor labels, codes, factor loadings, and Cronbach α.

Code and code names			Factor loading		Cronbach α
**Factor 1: Service and Communication Effectiveness**					0.97
	C01: Communication Issues	0.95			
	C05: Service Quality and Professionalism	1.00			
	C08: Doctor's Behavior and Tardiness	0.51			
	C15: Customer Service Training	0.97			
	C20: Language and Communication Barriers	0.60			
	C22: Staff Responsiveness	0.98			
	C26: Departmental Coordination	0.71			
	C27: Front Desk Service	0.85			
	C31: Organizational Efficiency	0.80			
	C33: Staff Attitude	0.98			
**Factor 2: Clinical Care and Patient Experience**					0.92
	C09: Nursing Care	0.77			
	C10: Patient Safety and Hygiene	0.64			
	C11: Electronic Health Info Management	0.47			
	C17: Diagnosis and Treatment	0.86			
	C21: Patient Rest and Comfort	0.53			
	C25: Medication Issues	0.65			
	C32: Doctor Changes and Qualifications	0.72			
**Factor 3: Facilities and Amenities Quality**					0.90
	C07: Facility Maintenance	0.61			
	C14: Cafeteria and Facilities	0.89			
	C16: Room and Amenities	0.87			
	C30: Hospital Facilities and Food Quality	0.71			
	C35: Lift and Equipment Issues	0.46			
	C41: Amenities Adequacy	0.70			
**Factor 4: Appointment and Patient Flow**					0.89
	C03: Appointment System Inconsistency	0.42			
	C04: Waiting Time	0.82			
	C13: Test and Result Processing	0.50			
	C28: Punctuality	0.76			
**Factor 5: Financial and Insurance Management**					0.88
	C06: Financial Concerns	0.89			
	C18: Insurance Issues	0.76			
	C36: Insurance Billing Mistakes	0.83			
**Factor 6: Patient Rights and Accessibility**					0.61
	C23: Patient Privacy	0.50			
	C24: Accessibility for Individuals with Disabilities	0.71			
	C29: Breastfeeding Support	0.45			

## Discussion

### Principal Findings

The analysis of review content identified a substantial portion of evaluations (n=12,035, 81%) accompanied by comments, with 19% (n=2903) lacking comments. Of the reviews with comments, 9.3% (n=1121) were deemed fake and excluded, leaving 9594 evaluations without issues and 1279 with issues. The use of the GPT-4o mini model for coding these reviews showed high interrater reliability, with an average Cohen κ score of 0.81, indicating strong agreement between human and AI coders. This high level of consistency supports the validity of the coding process and the reliability of the insights derived from the data.

### Granularity Issues

We acknowledge the lack of granularity of the codes produced by the GPT-4o mini model, that is, some of the codes are subsets of the other, and some of the codes contain more than 1 topic, making it nongranular. We noticed these results in the early phase of our research and decided to move forward with these codes. The first reason was that, in this study, we used inductive coding rather than deductive coding. Inductive coding is when codes and codebooks are created from scratch based on the available data (reviews), while deductive coding, on the other hand, is developing codebooks and coding the text using established concepts or theories. We did inductive coding and intentionally gave the freedom to the LLM to decide on the codes during codebook formation.

The second reason was that LLM was trained on a vast body of knowledge, which is not limited to just Medicine and Management. This includes Politics, Economics, Social, Technology, Legal, Environment, and Demographic domains (and this list is not exhaustive). Thus, the knowledge of LLM is not restricted to one or two theories only. Since the knowledge of LLM is cross-disciplinary, there are possibilities that we humans could not see the relevance of a code that is understandable only to the AI (LLM). This phenomenon is called the black box problem, in which mechanisms leading to an outcome are difficult to explain to humans but are effective in determining the outcome [56].

Despite the granularity issues, LLM has proven that the whole process is effective and reliable. Despite some of the codes looking nongranular to us humans, the LLM effectively uses the codes to label each review consistently across our data. For that reason, we were able to get excellent Cronbach α in almost all factors during factor analysis. If the lack of granularity causes confusion to the LLM itself, the coding phase will be inconsistent or chaotic and will then result in poor factor analysis. This is proof that the producer of the codes (the LLM itself) does understand the codes precisely, despite some of them looking nongranular to us humans.

Other than that, the freedom given to LLM to decide codes without any human intervention will also make it possible for us to replicate the system in the future without human intervention using the same pipeline. Thus, this kind of analysis can be adapted instantaneously to any industry and any web-based review platform that holds web-based review data. High replicability will make this study’s impact greater.

### Factor Analysis

Factor analysis identified 6 interpretable latent factors: “Service and Communication Effectiveness,” “Clinical Care and Patient Experience,” “Facilities and Amenities Quality,” “Appointment and Patient Flow,” “Financial and Insurance Management,” and “Patient Rights and Accessibility.” These factors encompass the key areas influencing patient satisfaction, as reflected in the items and their factor loadings. The cumulative explained variance for the seven variables is 0.74.

As we add (or remove) factors, we face a tradeoff between cumulative explained variance, reliability measured by Cronbach α, and interpretability. Adding more factors increases the cumulative explained variance but compromises Cronbach α on the additional factors and their interpretability. Removing factors, on the other hand, maintains Cronbach α high on all remaining factors together with interpretability, but reduces cumulative explained variance. Since any trade-off requires a decision maker to decide based on preference [57], we find it helpful to explain our preference below.

We prefer interpretable factors over higher explained variance because, unlike ML algorithms—for example, artificial neural networks, which can process the entire dataset to predict outcomes without fully understanding the underlying mechanisms—we humans emphasize understanding the factors behind observed variables as a base for future studies. The need for us to understand the mechanisms behind predictions is justified by Vamathevan et al [58], who raised concerns about the black box phenomenon in ML algorithms, how it leads to a lack of mechanism understanding, and mistrust. The cumulative variance of 0.74 for our 6 factors is considered very good since this study is behavioral, as noted by Williams et al [55].

### Implications

The themes we identified using web-based reviews, GPT-4o mini model, and factor analysis show strong alignment with global patterns identified in previous research. The systematic review of health care quality literature by Ferreira et al [59] revealed similar priorities in patient satisfaction assessment. Their analysis showed medical care appearing in 34% of studies, communication in 31%, doctor’s characteristics in 28%, accommodations in 23%, admission and discharge in 13%, nurse characteristics in 11%, appointment in 8%, environment in 8%, medical expenditure in 8%, and organization in 8%. The prominence of service quality and communication factors in our analysis mirrors these global patterns, though with some notable differences. For instance, our findings suggest a higher emphasis on appointment and waiting time issues in the Malaysian private health care context.

The high reliability of Service and Communication Effectiveness (α=0.97), coupled with strong factor loadings (0.51-1.00), demonstrates how tightly interconnected communication and service quality are in health care delivery. The strong correlation between communication issues and staff responsiveness (*r*=0.78) suggests that these aspects cannot be effectively addressed in isolation. This aligns with previous findings about the integrated nature of health care service quality (Ferreira et al [59]).

The emergence of distinct factors for both operational aspects (Appointment and Patient Flow, α=0.89) and support services (Financial and Insurance Management, α=0.88) indicates that patient satisfaction in private hospitals extends beyond clinical care. The moderate correlation between waiting time and staff attitude (*r*=0.41) reveals how operational inefficiencies can impact interpersonal aspects of care delivery. The questionable reliability of the Patient Rights and Accessibility factor (α=0.61) points to potential measurement challenges in these areas, suggesting a need for refined assessment tools. This finding reflects the broader challenge in health care research of quantifying and standardizing measurements of patient rights and accessibility (Williams et al [55]).

### Limitations

Despite providing valuable insights into patient experiences, this study has several important limitations that should be considered when interpreting the results. First, the reliance on web-based reviews as the primary data source may introduce sampling bias, as this method potentially excludes perspectives from patients who are less likely to share feedback on web-based review platforms, particularly older patients or those with limited digital access. While more granular categorization could have been achieved through human modulation, which might have improved the specificity and interpretability of our analysis, it also limits the true potential of LLMs, which are trained on vast bodies of knowledge. Future research could compare LACA outcomes based on codebooks entirely produced by LLMs versus those modulated by humans.

Second, the geographical scope of this study, being limited to private hospitals in Selangor, Malaysia, may not fully represent patient experiences in other regions or health care settings. This regional focus, while providing depth in local context, limits the generalizability of our findings to other geographical areas or health care systems. Third, variations in review platforms and user demographics could affect the comprehensiveness of our findings. Different web-based review platforms may attract distinct user demographics and encourage varying styles of feedback, potentially skewing our understanding of patient experiences [9]. Future research should address these limitations by incorporating multiple data sources, expanding geographical coverage, and considering diverse patient populations to provide a more comprehensive understanding of hospital quality issues.

### Conclusions

This study underscores the effectiveness of LACA for processing large-scale patient feedback, achieving high reliability (κ=0.81) between human and automated content analysis. The identification of six distinct factors explaining 74% of the variance provides a structured framework for understanding patient satisfaction in private hospitals.

Our findings suggest several actionable improvements for hospital management: (1) investing in integrated staff training programs focusing on communication skills and service delivery, given the strong factor loadings in these areas; (2) implementing advanced appointment systems and patient tracking technologies to address waiting time concerns, which appeared in 29.9% of negative reviews; and (3) developing integrated financial service units to handle billing, insurance, and payment issues cohesively, addressing the high-severity impact of these issues when they occur.

For policy makers, our results indicate the need for more structured guidelines in patient rights and accessibility standards. Health care regulators might consider developing comprehensive frameworks that address these aspects more systematically. Future research should expand this analysis to other geographical regions and health care contexts. Additionally, investigating the long-term impact of implementing LACA on web-based reviews could provide valuable insights into their effectiveness for continuous quality improvement in health care settings.

## Appendix

Multimedia Appendix 1 API (application programming interface) call for detecting presence of issue(s) in an online review.

Multimedia Appendix 2 API (application programming interface) call for induction of thematic codes from a list of 200 random reviews.

Multimedia Appendix 3 API (application programming interface) calls for justifying inclusion of each thematic code to develop a codebook.

Multimedia Appendix 4 Sample of actual online comments and the thematic codes assigned by the large language model.

Multimedia Appendix 5 Codebook for guiding content analysis.

Multimedia Appendix 6 Frequency of mentions of each thematic code.

Multimedia Appendix 7 Full list of cumulative explained variance from factor analysis.

## Abbreviations

AI: artificial intelligence
API: application programming interface
CV2: computer vision
HCAHPS: Hospital Consumer Assessment of Healthcare Providers and Systems
LACA: large language model–assisted content analysis
LLM: large language model
ML: machine learning
NLP: natural language processing

## Data Availability

The datasets analyzed during the factor analysis phase of this study are available in Google Drive [60]. The datasets generated during the large language model–assisted content analysis phase of this study are not made publicly available because they may contain sensitive information (name of hospitals or doctor) but are available from the corresponding author upon reasonable request or from public web-based review platforms at any time.
