# Supplementary material for: Analyzing Patient Complaints in Web-Based Reviews of Private Hospitals in Selangor, Malaysia, Using Large Language Model–Assisted Content Analysis: Mixed Methods Study
Source: JMIR Form Res. 2025 Jun 27;9:e69075. doi: 10.2196/69075 (PMC12254706; doi:10.2196/69075)
Supplement: Multimedia Appendix 1 [file formative_v9i1e69075_app1.docx]

def identify_issue(review):

# Initialize the OpenAI API client

client = OpenAI(

# This is the default and can be omitted

api_key= API_KEY

)

chat_completion = client.chat.completions.create(

messages=[

{

"role": "user",

"content": (

f"Does the following statement contain any "

f"issues:\n\nStatement:\n'{review}'\n\n"

f"Answer only Yes or No"

)

}

],

model="gpt-4o-mini",

)

r = chat_completion.choices[0].message.content.lower().strip()

return r # Result
