# Supplementary material for: Analyzing Patient Complaints in Web-Based Reviews of Private Hospitals in Selangor, Malaysia, Using Large Language Model–Assisted Content Analysis: Mixed Methods Study
Source: JMIR Form Res. 2025 Jun 27;9:e69075. doi: 10.2196/69075 (PMC12254706; doi:10.2196/69075)
Supplement: Multimedia Appendix 2 [file formative_v9i1e69075_app2.docx]

def identify_codes(sample_200):

# Initialize the OpenAI API client

client = OpenAI(

# This is the default and can be omitted

api_key= API_KEY

)

chat_completion = client.chat.completions.create(

messages=[

{

"role": "user",

"content": (

f"You are a thematic analyst, "

f"Now you need to produce thematic codes "

f"based on the following issues:\n\n"

f"Issues:\n{sample_200}\n\n"

f"Please list the thematic codes "

f"without explanation"

)

}

],

model="gpt-4o" # gpt-4o for more complex work

)

r = chat_completion.choices[0].message.content.strip()

return r # Result
