# Supplementary material for: Analyzing Patient Complaints in Web-Based Reviews of Private Hospitals in Selangor, Malaysia, Using Large Language Model–Assisted Content Analysis: Mixed Methods Study
Source: JMIR Form Res. 2025 Jun 27;9:e69075. doi: 10.2196/69075 (PMC12254706; doi:10.2196/69075)
Supplement: Multimedia Appendix 3 [file formative_v9i1e69075_app3.docx]

def identify_scale(review, codebook_file):

list_of_codes = open_txt(codebook_file).split('\n')

# Initialize the OpenAI API client

client = OpenAI(

# This is the default and can be omitted

api_key= API_KEY

)

result_list = []

for code in list_of_codes:

chat_completion = client.chat.completions.create(

messages=[

{

"role": "user",

"content": (

f"Given Likert scale 0 = 'Not an issue', "

f"1 = 'A small issue', "

f"2 = 'A moderate issue', "

f"3 = 'A serious issue', "

f"4 = 'An extremely serious issue', "

f"is '{code}' one of the issue(s) inside "

f"the following statement?\n\nStatement:\n"

f"{review}\n\nAnswer the value of the "

f"likert scale i.e 0 or 1 or 2 or 3 or 4"

)

}

],

model="gpt-4o-mini",

)

result_list.append(

chat_completion.choices[0].message.content.lower().strip()

)

return result_list
