# Supplementary material for: Analyzing Patient Complaints in Web-Based Reviews of Private Hospitals in Selangor, Malaysia, Using Large Language Model–Assisted Content Analysis: Mixed Methods Study
Source: JMIR Form Res. 2025 Jun 27;9:e69075. doi: 10.2196/69075 (PMC12254706; doi:10.2196/69075)
Supplement: Multimedia Appendix 4 [file formative_v9i1e69075_app4.docx]

| **No.** | **Actual Online Comment** | **Code(s) Assigned by LLM** |
| --- | --- | --- |
| **1** | Staff R****i very helpful but waiting time is too long^1^. 2 hours though. I’m patient no. 4. | 1. Waiting Time |
| **2** | Very friendly staff. We were the regular there since my new born daughter always went there check up and vaccines. It’s fine. But when comes to serious illness, something emergency^2,3^, they are really lack of experienced staff^1,4^. | 1. Work Load 2. Emergency Services 3. Patient Safety and Hygiene 4. Doctor’s Qualification and Doctor’s Change |
| **3** | On 2^nd^ Feb 2023 this S**a nurse said will check for me for an available appointment for this specialist doctor, whom I want to see. She didn't revert back to me at all^1,2,3,4^. I called again on 28^th^ February this nurse A***a said my appointment was slot on 23^rd^ March @ 5 pm so on 22^nd^ March I call up to confirmed and they said my name was not in the system and I have been waited for almost 1 month. Nurse M*****h help me to rebooked but didn't inform me that the date has been postponed to 24^th^ March^1,2^ instead she told me is 3 pm so I thought is in 23^rd^ March at 3 pm. Such a private hospital so incompetent and inefficient^4^ the nurses here. I just wants to make an appointment as this doctor specialist is always full. Simple tasks can't do it well. How you expect people will come to this hospital? | 1. Communication 2. Staff Responsiveness 3. Staff Attitude 4. Inefficient and Disorganized Processes |
| **4** | To operation director of A**C, you guys really need to improve the admission process^2^, because I never seen the improvement of this important section after multiple times visited here: 1) To do a 3 seconds Covid test, patient need to wait for 30 minutes although only 3 patients in queue^1^ 2) To sign 3 pages admission document, need to wait for 25 minutes. I saw only 2 staffs multitasking and very busy^3^, you guys should hire more staff, not to let your client waiting and suffering in pain 3) You guys should stay overnight in the room and use all the facilities in room / bathroom of hospital that you working now, you would find out what extremely lack of and what need urgently improve^4^. | 1. Waiting Time 2. Admission and Discharge Processes 3. Work Load 4. Communication |
| **5** | The worst and slowest hospital in the world^1^ took so long to admit patient^2^, even got deposit requested to customer emergency but not act like it avoid at all cost! Doctor don’t really properly check no dressing or cleaning the wounded area^4^, all they know only to charge customer go somewhere else, trust me counter only got one person, patient was left without attended by anyone^5^ to ask if feel uncomfortable and painful have to keep asking can i have that, can you provide this and that^3,5^ | 1. Waiting Time 2. Admission and Discharge Processes 3. Communication 4. Nursing Care 5. Customer Service |
| **6** | The environment is good, but the waiting time is a bit long.^1^ | 1. Waiting Time |
| **7** | Very bad customer service!^3,4^ Can’t even make an appointment^5^ with health screening department. They are not responsive at all^1,2^. | 1. Communication 2. Customer Service 3. Staff Responsiveness 4. Staff Attitude 5. Organizational Efficiency |
| **8** | Professional Doctors and nurses services is fantastic. However back end as in venepuncture, PIC (Person-In-Charge) is almost always not in her place. Pharmacy section should have more than 1 dispenser^1,3^. How can a premium hospital not have enough manpower^1^. Just to wait for medications is just too long.^2,3^ A**C is my favourite hospital but please improve on your support departments. | 1. Work Load 2. Waiting Time 3. Pharmacy Services |
| **9** | Overall were good. But need to improve service^2^ for E&R (Probably Emergency Room - ER) department. Waiting time a bit long^1^. Thank you. | 1. Waiting Time 2. Service Quality and Professionalism |
| **10** | Very bad arrangement in clinic Dr. A***r. Appointment set at 12 pm, but still need to wait for hours^1,2^. Understand they are patients need emergency care, but if that so, should not place the appointment half an hour later^1,2,3^, should be later so that we can come later. | 1. Inconsistent Appointment System 2. Waiting Time 3. Service Quality and Professionalism |
| **11** | Improve the pharmacy counter a little, the pharmacy cashier is not friendly^1,2^, I don't know if he is very arrogant, he is slow to take medicine^1,2^. Wait for more than 1 hour, because the medicine is left at the bottom, it's too late. Wait until there is no one at the pharmacy, keep on asking 5 - 6 times before looking for it, then you will get it^3^. | 1. Pharmacy Services 2. Staff Attitude 3. Inefficient and Disorganized Processes |
| **12** | My mum's medical check up on 21/12. 3 results pending and supposed to be sent via email to me^1^. Up until 27/12 still no email received until have to walk in myself^2^. Excuse given - due to public holidays. | 1. Test Processes and Results 2. Communication |
| **13** | Dear A**C, I was really disappointed with Ms. Wong's service from the Refund Department^1^. My son was released from the hospital on November 6^th^, 2022. It's been a month, and I'm supposed to get my refund in 14 days. I followed up on my refund status six times in three weeks and didn't receive any response until today^1,2^. I felt like I was begging the hospital for money. I finally called M****e on my own and questioning why I hadn't received my refund. M****e responded; "There are still questions from M****e that need to be answered by the hospital. The first question was sent on November 6^th^, and the hospital responded on November 11^th^. The second query was sent out on November 11th, and there has been no response as of today. M****e is still waiting for a response from the hospital"^1,2^. Please expedite the process. I am formally demanding my refund back by this Friday, 9^th^ December 2022. | 1. Finance (Consultation Fees, Billing and Refund) 2. Communication |
| **14** | Phone number given has nobody answer^1,2^, had been called for more than 10++ minute, operator keep telling our line was busy. | 1. Communication 2. Staff Responsiveness |
| **15** | I realised now a day hospital not really want entertain cash patients, hospital welcome those with medical card^1,3^ which they can order all sort of test and able charge without hassle^2,4^. No doubt this able help hospital generate more revenue. We believe private hospital can provide good advice in diagnosing sickness and given best solution, in reality not like this, very disappointing. | 1. Finance (Consultation Fees, Billing and Refund) 2. Service Quality and Professionalism 3. Customer Service 4. Doctor’s Behavior and Tardiness |
| **16** | Please save paper by issuing appointment letters via email or texts^1,3^. Nurse said must have paper because of scanning purposes. If airline tickets can go paperless with clear bar codes, I’m sure Assunta Hospital can invest in better scanners. Please don’t print OPA letters and save trees. We need them to tackle the CO_2_ emissions. Every little helps. Thanks. | 1. Organizational Efficiency 2. Facility Maintenance 3. Sustainable Practice |
| **17** | It's a shame. The hospital is good with caring and professional doctors. However your experience is marred by an absolutely useless patient management system^1,2,3^. It's always down which means the poor staff have to resort to manual tracking^2^. I would love to know who the vendor and IT manager are so I can avoid all places that use their system. | 1. Electronic Health Information Management 2. Inter-Departmental Coordination 3. Inefficient and Disorganized Processes |
| **18** | Delivery suite, lavender, and nursery department and Dr R**u blessing for us to be delivered. No regrets came to the right place and truly grateful. We are overwhelmed. But when comes to payment^2^ and pharmacy^3^ the worst service ever! Gotta wait extremely long^1^. | 1. Waiting Time 2. Finance (Consultation Fees, Billing and Refund) 3. Pharmacy Services |
| **19** | A good hospital overall. But please beware of the famous gastroenterologist there. He's the one with the most patients. Wins over his patients by being soft spoken. Gives his number to his patients but doesn't give updates, does not reply or just gives one word replies^1,2^. Likes to admit patients and treats them beyond his area of speciality. Doesn't update the family during the course of the admission^1^. Very evasive when I tried to see him after clinic hours. We went through hell when my parent was his patient. Regretted seeing him. I am sure there are better doctors around. Please dont be deceived by his demeanour. | 1. Communication 2. Doctor’s Behavior and Tardiness 3. Nursing Care |
| **20** | I had to request many times for the bag of diapers^1,2,3^ i was charged but the ward was using the packs i brought in. After many requests the missing bag of diapers appeared. Can i suggest accountability for the number of diapers used per day? This will settle any potential disputes^4^. And patients family are not made to feel degraded for constantly asking the already busy staff. | 1. Communication 2. Service Quality and Professionalism 3. Staff Responsiveness 4. Staff Attitude |
