# Supplementary material for: Analyzing Patient Complaints in Web-Based Reviews of Private Hospitals in Selangor, Malaysia, Using Large Language Model–Assisted Content Analysis: Mixed Methods Study
Source: JMIR Form Res. 2025 Jun 27;9:e69075. doi: 10.2196/69075 (PMC12254706; doi:10.2196/69075)
Supplement: Multimedia Appendix 5 [file formative_v9i1e69075_app5.docx]

| **Code** | **Label** | **Definition** |
| --- | --- | --- |
|  |  |  |
| **C01** | Communication Issues | Refers to any references or instances where difficulties, barriers, or misunderstandings in communication are mentioned or observed. These issues may involve misinterpretation of messages, lack of clarity, ineffective delivery, technological problems, language barriers, or any other factors that hinder clear and successful exchange of information between individuals or groups. This code captures both verbal and non-verbal communication challenges that disrupt effective interactions. |
| **C02** | Admission and Discharge Process | Refers to any references or instances related to the procedures, protocols, or experiences involved in admitting patients to and discharging them from a healthcare setting (e.g., hospital, clinic, or care facility). This includes the administrative steps, documentation, communication, coordination among staff, patient experiences, and any challenges or improvements related to the processes of admission and discharge. It captures both formal procedures and informal aspects, such as patient satisfaction, delays, confusion, or clarity of information shared with patients and their families. |
| **C03** | Appointment System Inconsistency | Refers to any references or instances where there are irregularities, discrepancies, or inefficiencies in the scheduling or management of appointments within a system. It includes issues such as scheduling errors, double bookings, delays, miscommunication about appointment times, lack of availability, or inconsistencies in how appointments are handled across different departments or staff members. This code captures frustrations, challenges, or disruptions experienced by patients, staff, or administrators due to a lack of coordination, organization, or reliability in the appointment system. |
| **C04** | Waiting Time | Refers to any references to the amount of time individuals (patients, clients, or visitors) spend waiting for services, appointments, or procedures. It encompasses both the perceived and actual duration of waiting, including waiting in various contexts such as for medical consultations, treatments, or administrative processes. This code captures negative experiences related to waiting time, including frustration, dissatisfaction, perceptions of inefficiency, or understanding of delays. It also includes mentions of waiting room conditions, communication regarding wait times, or efforts to reduce waiting time. |
| **C05** | Service Quality & Professionalism | Refers to any references or instances that describe the overall quality of services provided and the level of professionalism exhibited by staff or service providers. It includes aspects such as competence, responsiveness, attention to detail, courtesy, respect, and ethical behavior in interactions with patients, clients, or customers. This code captures negative assessments of service delivery, including factors such as staff knowledge, efficiency, communication skills, empathy, and the overall effectiveness of the service. It also encompasses the organizational culture or environment that influences how services are provided and how professionals conduct themselves. |
| **C06** | Financial Concerns | Refers to any references or instances that involve concerns, challenges, or issues related to the financial aspects of services or care. It includes topics such as the cost of services, affordability, insurance coverage, payment difficulties, billing disputes, or concerns about financial accessibility. This code captures expressions of stress, anxiety, or frustration related to the financial burden of receiving care or services, as well as any discussions on how financial constraints impact decision-making or access to necessary resources or treatment. |
| **C07** | Facility Maintenance | Refers to any references or instances related to the upkeep, cleanliness, functionality, and general condition of physical spaces and infrastructure within a facility. It includes mentions of issues such as broken equipment, poor sanitation, inadequate heating or cooling, maintenance delays, and overall safety or comfort concerns within the facility. This code captures negative feedback about the condition of the environment, as well as any impact that facility maintenance (or lack thereof) may have on the quality of service, patient or client experiences, and staff performance. |
| **C08** | Doctor's Behavior and Tardiness | Refers to any references or instances related to the conduct, demeanor, or punctuality of doctors within a healthcare setting. It encompasses aspects such as professionalism, communication style, empathy, attentiveness, and overall attitude toward patients, as well as behaviors that may affect the quality of care. It also includes issues related to tardiness or delays in doctor appointments, such as late arrivals, extended waiting times, or lack of communication regarding delays. This code captures negative perceptions of the doctor’s behavior and punctuality, and how these factors influence the patient experience. |
| **C09** | Nursing Care | Refers to any references or instances related to the care and services provided by nurses within a healthcare setting. It includes aspects such as the quality of patient interactions, the competency and professionalism of nurses, the responsiveness to patient needs, and the delivery of medical care, emotional support, and patient education. This code captures negative experiences of nursing care, highlighting areas such as attentiveness, communication, empathy, technical skills, and the overall impact of nursing care on the patient's comfort, safety, and wellbeing. |
| **C10** | Patient Safety and Hygiene | Refers to any references or instances related to the safety and cleanliness practices that directly impact the well-being of patients within a healthcare setting. It includes aspects such as infection control measures, sanitation of facilities and equipment, safe handling of medications and procedures, adherence to safety protocols, and overall hygienic conditions in patient care environments. This code captures negative feedback regarding the implementation of safety and hygiene standards, highlighting issues like cleanliness of rooms, availability of protective equipment, and the extent to which patient safety is prioritized to prevent harm or infections. |
| **C11** | Electronic Health Information Management | Refers to any references or instances related to the use, management, and accessibility of electronic health records (EHR) and other digital systems for storing, retrieving, and sharing patient health information. It includes aspects such as the accuracy, security, efficiency, and user-friendliness of electronic health information systems, as well as challenges or successes in managing patient data digitally. This code captures issues related to system integration, data privacy, technical difficulties, and the effectiveness of electronic systems in supporting clinical decision-making, communication between healthcare providers, and overall patient care. It also includes the impact of technology on patient confidentiality, data sharing, and healthcare workflow. |
| **C12** | Pharmacy Services | Refers to any references or instances related to the provision and quality of pharmaceutical care and services within a healthcare setting. It includes aspects such as the dispensing of medications, accuracy in prescription fulfillment, availability of medications, patient education on drug usage, and the interaction between pharmacists and patients or healthcare providers. This code captures negative experiences related to pharmacy services, including issues such as medication errors, wait times for prescriptions, clarity of medication instructions, and the overall professionalism and effectiveness of the pharmacy team in ensuring patient safety and satisfaction with their pharmaceutical care. |
| **C13** | Test and Result Processing | Refers to any references or instances related to the procedures and experiences involved in the ordering, handling, and communication of medical tests and their results within a healthcare setting. It includes aspects such as the accuracy, timeliness, and efficiency of test processing, as well as the clarity and delivery of test results to patients or healthcare providers. This code captures challenges or successes related to delays, miscommunications, errors in results, and the overall coordination between healthcare professionals, laboratories, and patients. It also encompasses patient experiences with understanding or interpreting their test results and any follow-up actions or guidance provided based on the results. |
| **C14** | Cafeteria and Facilities | Refers to any references or instances related to the quality, availability, and conditions of food services (cafeteria) and other on-site facilities within a healthcare or institutional setting. It includes aspects such as the variety, nutritional quality, affordability, and accessibility of food options, as well as the overall cleanliness, comfort, and functionality of facilities such as restrooms, waiting areas, and common spaces. This code captures negative feedback regarding the environment and services provided, and how they contribute to the overall experience of patients, visitors, or staff within the facility. |
| **C15** | Customer Service Training | Refers to any references or instances related to the training programs and initiatives aimed at enhancing the customer service skills of staff within an organization. It includes aspects such as the content, quality, and effectiveness of training in areas like communication, problem-solving, empathy, conflict resolution, and professionalism when interacting with clients, patients, or customers. This code captures discussions about the impact of customer service training on staff behavior, the consistency of service delivery, and overall satisfaction of those receiving the service. It may also include perceptions of whether staff are adequately trained to handle diverse customer needs and situations. |
| **C16** | Room and Amenities | Refers to any references or instances related to the condition, comfort, and features of patient rooms or service spaces, as well as the available amenities within a healthcare or hospitality setting. It includes aspects such as room cleanliness, furniture, bed comfort, lighting, privacy, temperature control, and the availability of essential amenities (e.g., toiletries, Wi-Fi, television, phone services). This code captures negative feedback regarding the physical environment and amenities provided, and how they contribute to the overall experience, comfort, and satisfaction of patients, clients, or visitors. |
| **C17** | Diagnosis and Treatment | Refers to any references or instances related to the processes of diagnosing and treating medical conditions within a healthcare setting. It includes aspects such as the accuracy and clarity of diagnoses, the methods and approaches used in treatment, the communication of diagnosis and treatment plans to patients, and the effectiveness of prescribed treatments or interventions. This code captures negative experiences related to how diagnoses are made, how treatment options are explained and delivered, and the overall satisfaction or dissatisfaction with the care provided. It may also include mentions of delays, challenges in treatment effectiveness, or patient understanding of their condition and treatment plan. |
| **C18** | Insurance Issues | Refers to any references or instances related to challenges, complications, or concerns involving health insurance within a healthcare setting. It includes issues such as coverage limitations, claim denials, billing errors, pre-authorization requirements, out-of-pocket costs, or confusion regarding insurance policies. This code captures both patient and provider experiences related to navigating insurance processes, including frustrations with reimbursement procedures, communication with insurance companies, or discrepancies between services rendered and insurance coverage. It also encompasses the impact of insurance-related issues on access to care, treatment decisions, and financial stress. |
| **C19** | Emergency Services | Refers to any references or instances related to the delivery, access, and quality of emergency care within a healthcare setting. It includes aspects such as response times, the efficiency of triage systems, the availability of emergency personnel and resources, and the quality of medical treatment provided during urgent or critical situations. This code captures negative experiences with emergency services, including patient perceptions of care speed, communication with healthcare providers, the professionalism of emergency staff, and the overall effectiveness of emergency interventions in managing acute health conditions or crises. |
| **C20** | Language and Communication Barriers | Refers to any references or instances related to challenges in communication due to language differences or other barriers that affect the exchange of information between individuals. It includes issues such as misinterpretations, misunderstandings, difficulty in conveying or receiving important information, and the need for interpreters or translation services. This code captures both verbal and non-verbal communication barriers that hinder effective interaction, including the impact on service delivery, patient care, decision-making, and overall satisfaction. It also encompasses any strategies or efforts to overcome these challenges, such as the use of technology, bilingual staff, or cultural sensitivity training. |
| **C21** | Patient Rest and Comfort | Refers to any references or instances related to the physical and emotional comfort of patients during their stay or treatment in a healthcare setting. It includes aspects such as the quality of rest (e.g., sleep, relaxation), comfort of the patient’s environment (e.g., bed, room temperature, noise levels), and the availability of amenities or support to enhance comfort (e.g., pillows, blankets, privacy). This code captures both the physical conditions that affect a patient’s ability to rest and recover, as well as the emotional and psychological support provided to ensure a sense of well-being. It may also include mentions of staff responsiveness to comfort needs, as well as the overall atmosphere of the healthcare setting in promoting patient comfort. |
| **C22** | Staff Responsiveness | Refers to any references or instances related to the speed, attentiveness, and effectiveness of staff in responding to the needs, concerns, or requests of patients, clients, or visitors within a healthcare or service setting. It includes aspects such as the timeliness of responses, the willingness to assist, the quality of interaction, and the efficiency with which staff address issues or provide support. This code captures negative experiences of how staff members (e.g., healthcare providers, administrative personnel, support staff) attend to and communicate with individuals, as well as the impact of responsiveness on overall satisfaction and the quality of care or service provided. |
| **C23** | Patient Privacy | Refers to any references or instances related to the protection and respect of patient confidentiality and personal information within a healthcare setting. It includes aspects such as the safeguarding of medical records, private conversations, and any sensitive data shared between patients and healthcare providers. This code captures both the policies and practices that ensure patient privacy, as well as any concerns or breaches related to unauthorized access, disclosure of information, or lack of confidentiality. It also includes the ways in which healthcare staff uphold or violate privacy expectations, as well as patients’ perceptions of their privacy being respected throughout their care experience. |
| **C24** | Accessibility for Individuals with Disabilities | Refers to any references or instances related to the availability and quality of accommodations and services that ensure individuals with disabilities can access and navigate healthcare or service settings. It includes aspects such as physical accessibility (e.g., wheelchair ramps, elevators, accessible restrooms), communication accessibility (e.g., sign language interpreters, assistive technologies, accessible information formats), and the provision of specialized support for patients with various disabilities (e.g., mobility, sensory, cognitive). This code captures negative experiences related to how well a healthcare or service environment accommodates individuals with disabilities, as well as any challenges, barriers, or improvements in making the environment more inclusive and accessible. |
| **C25** | Medication Issues | Refers to any references or instances related to problems or challenges encountered with medications within a healthcare setting. It includes aspects such as medication errors (e.g., incorrect dosage, wrong medication), delays in receiving medications, issues with prescription fulfillment, side effects, misunderstandings about medication instructions, and difficulties in obtaining or affording prescribed medications. This code captures both patient and healthcare provider experiences related to medication management, including concerns about the effectiveness, safety, or accessibility of medications, as well as the impact of these issues on patient health and treatment outcomes. |
| **C26** | Departmental Coordination | Refers to any references or instances related to the communication, collaboration, and integration of services across different departments within a healthcare or organizational setting. It includes aspects such as the smooth flow of information between departments, the coordination of care or services, joint decision-making processes, and the timeliness and efficiency of inter-departmental collaboration. This code captures negative experiences related to how well departments work together to provide comprehensive, patient-centered care, and any issues arising from lack of coordination, delays, or fragmentation of services that impact patient outcomes or operational efficiency. |
| **C27** | Front Desk Service | Refers to any references or instances related to the quality of service provided by front desk staff or receptionists in a healthcare or service setting. It includes aspects such as the professionalism, friendliness, efficiency, and helpfulness of staff in managing patient or client inquiries, appointment scheduling, check-ins, and general administrative tasks. This code captures negative experiences related to the first point of contact within a facility, including issues such as long wait times, lack of communication, or difficulties in obtaining necessary information or assistance. It also reflects how the front desk service contributes to the overall patient or client experience and satisfaction. |
| **C28** | Punctuality | Refers to any references or instances related to the timeliness of appointments, services, or staff within a healthcare or service setting. It includes aspects such as the on-time arrival of healthcare providers, timely initiation of procedures or appointments, and adherence to scheduled times. This code captures negative experiences related to punctuality, including instances of delays, waiting times, and how such issues affect patient or client satisfaction, perception of service quality, and overall experience. It also reflects the impact of punctuality on the efficiency of operations and the delivery of care or services. |
| **C29** | Breastfeeding Support | Refers to any references or instances related to the assistance, guidance, and resources provided to mothers to support breastfeeding in a healthcare or community setting. It includes aspects such as the availability of lactation consultants, education on breastfeeding techniques, emotional support, advice on managing common breastfeeding challenges (e.g., latching, supply issues, pain), and access to relevant materials or equipment (e.g., pumps, pillows). This code captures negative experiences with breastfeeding support, highlighting the effectiveness of the support offered, the professionalism and empathy of healthcare providers, and the impact on the mother's confidence, comfort, and success with breastfeeding. |
| **C30** | Hospital Facilities and Food Quality | Refers to any references or instances related to the physical environment of the hospital and the quality of food provided to patients and visitors. It includes aspects such as the cleanliness, comfort, and functionality of hospital facilities (e.g., patient rooms, waiting areas, restrooms, common spaces), as well as the variety, nutritional value, taste, and overall quality of food served. This code captures negative feedback about the hospital’s infrastructure and food services, highlighting how these factors contribute to the overall patient experience, comfort, satisfaction, and well-being during their stay or visit. |
| **C31** | Organizational Efficiency | Refers to any references or instances related to the effectiveness and streamlined operation of an organization or healthcare setting. It includes aspects such as the smoothness of internal processes, timely delivery of services, coordination among staff, resource management, and the ability to minimize delays or disruptions. This code captures negative experiences regarding how well the organization utilizes its resources, manages workflows, and ensures that services are delivered in an organized, prompt, and cost-effective manner. It also reflects the impact of organizational efficiency on patient care, staff performance, and overall satisfaction. |
| **C32** | Doctor Changes and Qualifications | Refers to any references or instances related to changes in healthcare providers (e.g., doctor substitutions, staff turnover) and the qualifications or expertise of doctors within a healthcare setting. It includes aspects such as the introduction of new doctors, changes in the assigned physician, transitions between healthcare providers, and the qualifications, experience, or specialization of the doctors involved in patient care. This code captures negative experiences with doctor changes, including concerns about continuity of care, trust, and familiarity with the patient’s medical history, as well as the perceived competence or confidence in a doctor’s qualifications and their ability to provide effective care. |
| **C33** | Staff Attitude | Refers to any references or instances related to the behavior, demeanor, and general attitude of staff members within a healthcare or service setting. It includes aspects such as professionalism, empathy, friendliness, respect, and the overall approachability of staff, as well as their willingness to help or provide assistance. This code captures negative experiences regarding how staff interact with patients, clients, or visitors, and how their attitudes impact the overall experience. It may also encompass staff responsiveness, communication style, and the emotional tone conveyed during interactions. |
| **C34** | Staff Overload | Refers to any references or instances related to staff being overburdened or stretched beyond their capacity to effectively perform their duties within a healthcare or service setting. It includes aspects such as high patient or client caseloads, insufficient staffing levels, excessive work hours, or the pressure of managing multiple responsibilities. This code captures both the impact of staff overload on the quality of care or service, as well as any associated challenges such as burnout, delays, decreased efficiency, and staff stress. It also reflects how staff overload may affect patient or client experiences, including longer wait times, reduced attention, or diminished service quality. |
| **C35** | Lift and Equipment Issues | Refers to any references or instances related to problems or challenges with the functionality, maintenance, or availability of lifts (elevators) and other essential equipment within a healthcare or service setting. It includes aspects such as malfunctioning lifts, delays in lift access, inadequate equipment for patient handling (e.g., wheelchairs, stretchers, medical devices), and the overall reliability of tools necessary for efficient patient care and service delivery. This code captures both the impact of equipment failures on operational efficiency and patient experience, as well as the staff’s ability to provide safe, timely, and effective care. |
| **C36** | Insurance Billing Mistakes | Refers to any references or instances related to errors or discrepancies in the billing process involving insurance claims. It includes issues such as incorrect charges, misapplied insurance coverage, claim denials, duplicate charges, billing for services not rendered, or delays in processing claims. This code captures both the impact of these mistakes on patients (e.g., unexpected out-of-pocket costs, confusion) and the administrative challenges faced by healthcare providers or insurance companies in rectifying billing issues. It also reflects patient or client frustration, the need for clarification, and any efforts to resolve billing errors. |
| **C37** | Room Readiness | Refers to any references or instances related to the preparation and condition of patient rooms or service areas prior to patient use within a healthcare or service setting. It includes aspects such as the cleanliness, availability of necessary equipment, proper functioning of room amenities (e.g., lighting, temperature control, medical devices), and overall readiness for patient care. This code captures negative experiences regarding how well rooms are prepared and maintained, as well as any delays, deficiencies, or inconveniences caused by rooms not being adequately ready for use at the time of patient arrival. |
| **C38** | Workplace Rushing | Refers to any references or instances related to the hurried or pressured environment in a workplace, where employees or service providers feel compelled to work quickly or inefficiently due to time constraints, high workloads, or organizational demands. It includes aspects such as employees rushing through tasks, interactions, or processes, potentially compromising the quality of work, attention to detail, or service delivery. This code captures both the causes of workplace rushing (e.g., understaffing, tight deadlines) and its impact on staff performance, employee stress, patient care, or customer satisfaction. It may also reflect how the rushed environment affects workplace morale and communication. |
| **C39** | Comparative Evaluation | Refers to any references or instances where individuals compare or evaluate the experiences, behaviors, or characteristics of the Chinese, Indian and Malay communities, particularly within a specific context (e.g., healthcare, education, workplace, or cultural practices). It includes aspects such as perceived differences in values, practices, communication styles, or treatment preferences between the two groups. This code captures negative evaluations, discussions, or judgments about how these communities interact with services, systems, or each other, and the implications of such comparisons on societal attitudes, cultural understanding, or service delivery. It may also reflect issues related to stereotypes, biases, or efforts to promote inclusivity. |
| **C40** | Inefficient and Disorganized Processes | Refers to any references or instances related to processes, systems, or workflows that are perceived as ineffective, disorganized, or lacking in structure within a healthcare or service setting. It includes aspects such as delays, confusion, poor coordination, redundant steps, lack of clear procedures, or the failure to streamline operations. This code captures both the direct impact of inefficiency and disorganization on service delivery, patient care, or customer experience, as well as staff frustrations or challenges in navigating such processes. It may also reflect suggestions for improvement or experiences of frustration due to disorganized environments that hinder productivity and satisfaction. |
| **C41** | Amenities Adequacy | Refers to any references or instances related to the sufficiency and quality of amenities provided within a healthcare or service setting. It includes aspects such as the availability, functionality, comfort, and overall appropriateness of amenities like seating, restrooms, food services, parking, Wi-Fi, and other facilities provided for patients, clients, or visitors. This code captures negative experiences regarding whether the amenities meet the needs of individuals and contribute to their overall satisfaction and comfort. It may also reflect any gaps in amenities that impact the experience of care or services provided. |
