# Supplementary material for: Analyzing Patient Complaints in Web-Based Reviews of Private Hospitals in Selangor, Malaysia, Using Large Language Model–Assisted Content Analysis: Mixed Methods Study
Source: JMIR Form Res. 2025 Jun 27;9:e69075. doi: 10.2196/69075 (PMC12254706; doi:10.2196/69075)
Supplement: Multimedia Appendix 7 [file formative_v9i1e69075_app7.docx]

F01: 0.50, F02: 0.59, F03: 0.64, F04: 0.69, F05: 0.71, F06: 0.74, F07: 0.76, F08: 0.77, F09: 0.79, F10: 0.80, F11: 0.81, F12: 0.82, F13: 0.84, F14: 0.85, F15: 0.86, F16: 0.87, F17: 0.88, F18: 0.89, F19: 0.90, F20: 0.90, F21: 0.91, F22: 0.91, F23: 0.92, F24: 0.93, F25: 0.95, F26: 0.96, F27: 0.96, F28: 0.97, F29: 0.98, F30: 0.99, F31: 0.99, F32: 1.00, F33: 1.00, F34: 1.00, F35: 1.00, F36: 1.00, F37: 1.00, F38: 1.00, F39: 1.00, F40: 1.00, F41: 1.00.

Note: F = Latent Factor
